# The association between symptomatic remission and social support in community-dwelling schizophrenia patients during COVID-19

**DOI:** 10.3389/fpsyt.2024.1404059

**Published:** 2024-06-27

**Authors:** Lansicheng Yao, Hongying Liu, Qiuyu Pan, Xiaobing Tian

**Affiliations:** ^1^ Foreign Affairs Office, North Sichuan Medical College, Nanchong, Sichuan, China; ^2^ Department of Psychiatric Rehabilitation, Nanchong Psychosomatic Hospital Affiliated to North Sichuan Medical College, Nanchong, Sichuan, China; ^3^ Teaching and Research Office of Social Medicine and Health Management, School of Public Health, North Sichuan Medical College, Nanchong, Sichuan, China; ^4^ School of Medicine, Tibet University, Lhasa, Tibet, China; ^5^ Department of Epidemiology and Health Statistics, School of Public Health, North Sichuan Medical College, Nanchong, Sichuan, China

**Keywords:** COVID-19, schizophrenia, symptomatic remission, social support, community

## Abstract

**Introduction:**

Schizophrenia is a severe and enduring psychiatric disorder, characterized by substantial challenges in achieving symptomatic remission. Up to now, there have been limited studies examining the association between remission status and social support in patients with schizophrenia during COVID-19. This study aimed to investigate the remission status of community-dwelling schizophrenia patients during the pandemic and explore whether symptomatic remission is significantly associated with social support in a cross-sectional study.

**Methods:**

727 schizophrenia patients were recruited using a cluster random sampling method in the local community. Face-to-face interviews were used to gather data on participants’ clinical characteristics, sociodemographic, social support, and symptomatic remission criteria. Logistic regression models were deployed to identify potential relationships between symptomatic remission and social support.

**Results:**

Among the 727 patients in our study, a substantial proportion of 522 (71.80%) achieved symptomatic remission, while 205 (28.2%) did not. Remarkably, those who achieved symptomatic remission exhibited a higher level of social support (28.32 ± 6.82) compared to those who did not. The proportion of patients achieving symptomatic remission in the low (19.4%), medium (46.2%), and high (34.3%) social support groups was 56.7%, 66.1%, and 88.0%, respectively. Moreover, the crude odds ratio for the association between social support and symptomatic remission was 3.20 (95% CI: 2.45–4.18). After controlling for all confounding factors, the adjusted odds ratio remained significant at 3.02 (95% CI: 2.30–3.97).

**Discussion:**

This consistent association underscores the critical role of social support in influencing symptomatic remission among community-dwelling schizophrenia patients, especially during the COVID-19 pandemic. Reinforcing the utilization of social support for fostering symptomatic remission among individuals with schizophrenia who reside in the community during such crises is recommended.

## 1 Introduction

Schizophrenia is a severe form of mental illness causing adverse functional, behavioral, and social deficits in those affected (1). The lifetime prevalence of Schizophrenia is about 1% (21 million) and 0.7% (8 million) globally and in China, respectively (2, 3). In China, over 90% of schizophrenia patients are now living in the community rather than in a psychiatric institution (4). Regular follow-up and continuity of care are therefore provided at the community level for these patients (5).

Symptomatic remission is the first step toward recovery for patients with schizophrenia (6). According to the Remission in Schizophrenia Working Group (RSWG), remission is achieved when positive and negative symptoms are adequately controlled (7). The operational criterion by RSWG is based on the severity of eight core psycho-pathological symptoms as assessed by the Positive and Negative Symptoms Scale (PANSS) score, which defines that each item does not exceed a score of mild for at least 6 months or more (8). The specific criteria by RSWG substantially enhanced the comparability of different studies and facilitated the effectiveness of predictors screening. However, the reported remission rates in patients with schizophrenia by previous studies varied widely, depending on the study design and target population. The lack of consistent findings hampers the efforts to suggest effective intervention strategies for this population. Researchers hence emphasized the need for more insights into potential attributes of stable remission so as to formulate appropriate rehabilitation plans for these patients.

Although a number of factors may impact remission in schizophrenia patients, social support as a protective factor facilitating rehabilitation has been confirmed by the majority of studies (9, 10). Social support is postulated to serve as a modulating factor to alleviate the negative effects of environmental stressors, or buffering factors against psychological stressors like fatigue, depression, insomnia, and suicidal intention (11). This could help patients to adapt to living in the community, adhere to medication, and enhance self-efficacy and coping style as well as social engagement (12). This, therefore, favors better symptomatic evolution. However, given the diverse cultural context and level of self-acceptance, the concept of social support varied across regions. Validation and comparison of prior findings about social support across published studies would be less informative and misleading.

The global pandemic of coronavirus disease-19 (COVID-19), has had an unprecedented impact on populations around the world (13). Given the vulnerability to social determinants of health for schizophrenia patients, this may pose a disproportionate burden on them and have a profound impact on their rehabilitation (13, 14). During COVID-19, some public health strategies to contain the spread of the disease like social distancing and social isolation have been widely implemented. This made it hard to conduct in-person contacts in the community and in patients’ homes, and led to dramatic changes to their social networks and style of interpersonal communications. However, there are few studies examining its impact on social support during this period and its relevance to symptomatic remission. In the present study, we aim to, 1) describe the social support level and symptom remission status during COVID-19, and 2) explore the impact of social support on symptom remission in community-dwelling schizophrenia patients.

## 2 Methods

### 2.1 Study setting and design

A cross-sectional study was conducted in Yingshan, Sichuan Province between December 2020 and July 2021. The study was approved by the Institutional Ethics Review Board (IERB) of the Psychosomatic Hospital Affiliated to North Sichuan Medical College in June 2021. Written informed consent was obtained from all participants before they began the questionnaire.

In mainland China, the management of severe mental disorder patients follows a centralized approach. Diagnosed patients with severe schizophrenia are registered and tracked on the National Severe Mental Disorders Information System platform, undergoing subsequent follow-ups (15). The study population consisted of registered patients of the Sichuan Province Comprehensive Management Information Platform for Severe Mental Disorders (16, 17), with 2,297 individuals registered as of December 31, 2020, resulting in a reported prevalence rate of 0.37%.

### 2.2 Participants

Participants were included if they met the following criteria: (1) Diagnosed severe mental health patients are registered on the Sichuan Province Comprehensive Management Information Platform (16); (2) age between 16 and 85 years; (3) community management at home for ≥6 months. Patients who were in an unstable condition such as incapacitated or unable to complete the survey, and those who had other mental or serious physical illnesses severe enough to negatively affect the authenticity of the study data, and those who had daycare centers, the workplace for people with mental illness, visiting nursing, were excluded due to decreased access to healthcare services and changes in medication adherence resulting from lock-downs.

### 2.3 Symptomatic remission

The Positive and Negative Syndrome Scale (PANSS) was used to assess three subscales of schizophrenia symptoms (positive, negative, and general psychopathology) (7), with a total of 30 items and 3 supplementary items to assess the risk of assault. In the routine management of patients with severe mental illness in China, the PANSS is a fundamental tool for monitoring the severity of patient symptoms (18). The scale, primarily used in adults, was evaluated by a psychiatrist during the psychiatric examination of schizophrenia patients and combined clinical examination with the information provided by patients’ caregivers. The evaluation took between 30 and 50 minutes. To improve the scale’s objectivity and standardization, the authors created a clinical syllabus for the PANSS (SCI-PANSS), which examiners could use as a reference. Each PANSS item had a definition as well as a specific 7-point operational rating scale based on increasing levels of psychopathology, with the severity of symptoms graded on a scale of 1 (none) to 7 (overwhelming) (19). The Cronbach’s alpha for PANSS in the present study was 0.79, indicating good internal consistency.

According to the RSWG, remission is achieved when positive and negative symptoms are adequately controlled (7). The operational criterion by RSWG is based on the severity of eight core psycho-pathological symptoms as assessed by the PANSS score, which defines that each item does not exceed a score of mild for at least 6 months or more (8). Therefore, in this study, symptomatic remission was defined as a score of 3 or lower on all relevant PANSS items, in accordance with these criteria.

### 2.4 Social support

The Social Support Rating Scale (SSRS) is a self-rated instrument developed by Xiao Shuiyuan (20, 21) and is one of the most widely used tools for assessing social support in China. Some studies have demonstrated good reliability and validity of this method in patients with severe mental illness in China (21). The SSRS is divided into three categories: subjective support, objective support, and support utilization, with a total score of 40, including items 1, 3, 4, 5 for subjective support, items 2, 6, 7 for objective support, and items 8, 9, 10 for support utilization across three dimensions. A higher score indicates greater social support. The internal consistency of the scale was good, with a Cronbach’s alpha of 0.784. In this study, we calculated scores for the three social support subgroups based on the remission status of schizophrenia patients, namely PANSS criteria-positive, negative, and general psychopathology symptoms domains, as well as the total scale and three subscales.

### 2.5 Sociodemographic characteristics and related variables

Sociodemographic characteristics such as age, gender, marital status, education level, residential area, duration since diagnosis, and duration of untreated psychosis as well as subject-related variables such as family income, family members, and family caregivers’ information, were collected by the same interviewers through direct consultation. Family economic status was classified into three levels: low, medium, and high, based on the participants’ self-assessment criteria. Individual interviews were conducted with patients and their caregivers to ensure their privacy and confidentiality during the data collection process.

### 2.6 Study procedure and data collection

Patients’ original background information was gathered from a dataset shared with another study (14) that sought to validate the PANSS and SSRS results. The sample size was calculated based on the formula: $\text{n}={\left(\frac{{\text{z}}_{1}-a/2}{\text{δ}}\right)}^{2}\times \text{p}\times (1-\text{p})$ . Using α=0.05, per the literature, a symptomatic remission rate of p=43%, a tolerance error *δ* = 4.3% (10% of the symptomatic remission rate), yielding 509 people subsequently. Considering a 30% missed visit rate, the final (minimum) sample size was 727 people. Based on the results, four zones in Yingshan County (1 urban and three rural regions, as defined by Chinese administrative classification) (15) were chosen using a cluster random sampling method, with the mental health service management section serving as a sampling unit. Finally, a total of 876 patients who met the study definition within the management ward were included as survey participants.

This survey was conducted during the partial reopening period of the COVID-19 pandemic. All participants signed a consent form before participation in the study and filled out a questionnaire composed of PANSS, SSRS, and sociodemographic characteristics with the researcher’s assistance item by item. For patients who are unable to respond independently due to factors such as low educational level, impaired vision, or limited mobility, the investigator will verbally present each item and conduct the survey through face-to-face interviews. The PANSS scales were scored by psychiatrists during patient face-to-face visits. The fee was offered to participants 20 CNY (equivalent to $2.8, November 7th, 2022). For roughly 10% of those who were unable to attend the scheduled location, the interview was conducted in the patients’ homes.

### 2.7 Statistical analysis

We performed data analysis using the SAS9.4 software version (SAS Inc., NCSU, USA). Descriptive statistics with means and standard deviations were used for continuous variables, while categorical variables were reported using frequency counts. The chi-squared test was employed to compare differences between the remission and non-remission groups.

The remission rate was calculated by dividing the total number of respondents by the number of symptomatic remission patients. To investigate the relationship between social support and symptomatic remission, three logistic regression models were developed. Model 1 displayed the crude odds ratio of social support and symptomatic remission with remission as the dependent variable (remission=1, non-remission=0). Model 2 demonstrated the adjusted odds ratio of Model 1 by controlling for patients’ sociodemographic characteristics. Model 3 included family-related variables. The social support level was the independent variable.

Finally, all covariates were included as confounders in the logistic regression analysis to validate the observed correlation between social support and symptomatic remission. Statistical significance was set at *P*< 0.05.

## 3 Results

### 3.1 Descriptive analyses

A total of 727 Schizophrenia Patients were enrolled in the present study. The mean age was 48.95± 12.33 years. As shown in **Table 1**, there were 285 (39.0%) males and 442(61.0%) females. The respondents were predominantly from rural areas (82.7%). The majority received education up to the primary level or below. About two-thirds of patients (67.1%) were married. About half of the patients had the disease more than 5 years after diagnosis. Of the 727 patients, 522(71.80%) achieved symptomatic remission while 205(28.2%) not. The univariate analysis showed that patient’s education level, disease duration since diagnosis, and family economic status were significantly associated with symptomatic remission.

**Table 1:** Socio-demographic Characteristics and Family-Related Factors among Schizophrenia Patients (N=727).

Variables	Total	Remission	No (n=205)	*x* ^2^	*p*
		Yes (n=522)			
Age				0.83	0.66
<40	180 (24.8)	134 (74.4)	46 (25.6)		
40–60	399 (54.9)	283 (70.9)	116 (29.1)		
>60	148 (20.3)	105 (70.9)	43 (29.1)		
Gender				3.68	0.06
Male	285 (39.0)	216 (75.8)	69 (24.2)		
Female	442 (61.0)	306 (69.2)	136 (30.8)		
Residential area				0.30	0.58
Urban	126 (17.3)	93 (73.8)	33 (26.2)		
Rural	601 (82.7)	429 (71.4)	172 (28.6)		
Education level				13.80	<0.01
Illiteracy or Semi-literacy	205 (28.2)	127 (62.0)	78 (38.0)		
Primary school	297 (40.8)	223 (75.1)	74 (24.9)		
Junior school or above	225 (31.0)	172 (76.4)	53 (23.6)		
Marital status				2.37	0.31
Non married	170 (23.4)	115 (67.7)	55 (32.3)		
Married	488 (67.1)	359 (73.6)	129 (26.4)		
Other	69 (9.5)	48 (69.6)	21 (30.4)		
Duration since diagnosis				5.88	0.05
<1 year	81 (11.4)	64 (79.0)	17 (21.0)		
1–5 years	298 (41.0)	222 (74.5)	76 (25.5)		
>=5 years	348 (47.9)	236 (67.8)	112 (32.2)		
Family economic status				10.56	0.01
Low	231 (31.8)	165 (71.4)	66 (28.6)		
Medium	442 (60.8)	308 (69.7)	134 (30.3)		
High	54 (7.4)	49 (90.7)	5 (9.26)		
Number of family members				0.60	0.74
<=2	204 (28.1)	144 (70.6)	60 (29.4)		
3–4	306 (42.1)	218 (71.2)	88 (28.76)		
>=5	217 (29.9)	160 (73.7)	57 (26.3)		
Primary caregiver				2.30	0.51
Parents	254 (34.9)	187 (73.6)	67 (26.4)		
Spouse	378 (52.0)	272 (72.0)	106 (28.0)		
Son/daughter	55 (7.6)	35 (63.6)	20 (36.4)		
Others	40 (5.5)	28 (70.0)	12 (30.0)		
Caregiver’s Education Level				3.13	0.21
Illiteracy or Semi-literacy	164 (22.6)	118 (72.0)	46 (28.1)		
Primary school	372 (51.2)	258 (69.4)	114 (30.7)		
Junior school or above	146 (76.44)	146 (76.4)	45 (23.6)		

*Other, including divorced, widowed, and unspecified categories. The bold P-value represents statistics with statistical significance.

Percentage means respondents/Total numbers; Rate means symptomatic remission patients/total respondents.

The SSRS scores are shown in **Table 2**. The overall social support score (28.32 ± 6.82) in the remission group was significantly higher than that (23.67 ± 4.89) in the non-remission group. Further comparison across three domains revealed that the subjective (17.88 ± 5.47) and objective (4.81 ± 1.53) domains in the remission group were significantly higher than the scores (14.09 ± 3.93) and (4.36 ± 1.40) in the non-remission group. Similarly, the score of the utilization domain in the remission group was higher than the corresponding scores in the non-remission group; however, no significant difference was observed between the two groups. See **Table 2**.

**Table 2:** Scores on Social Support Rating Scale among Schizophrenia Patients, by Remission Status (N=727).

Social Support	Total	Remission		*t*	*p*
		Yes (n=522)	No (n=205)		
Subjective	16.81 ± 5.36	17.88 ± 5.47	14.09 ± 3.93	9.06	<0.01
Objective	4.69 ± 1.51	4.81 ± 1.53	4.36 ± 1.40	3.36	<0.01
Utilization	5.51 ± 2.82	5.63 ± 2.82	5.22 ± 2.83	1.73	0.08
Overall	27.01 ± 6.67	28.32 ± 6.82	23.67 ± 4.89	8.90	<0.01

Bold P-value represents statistics with statistical significance.

According to the cut-off value of 20 and 30 on SSRS, there were 141(19.4%), 336(46.2%) and 250(34.4%) patients to be categorized as at low, medium, and high levels of social support. The remission rate in the three social support groups was 56.7%, 66.1%, and 88.0%, showing a significant difference across groups. See **Table 3**.

**Table 3:** Remission Rate among Schizophrenia Patients, by Social Support Level (N=727).

Social Support	Remission			*x* ^2^	*p*
	Total	Yes (n=522)	No (n=205)	53.65	<0.01
Low	141 (19.4)	80 (56.7)	61 (43.3)		
Medium	336 (46.2)	222 (66.1)	114 (33.9)		
High	250 (34.4)	220 (88.0)	30 (12.0)		

Bold P-value represents statistics with statistical significance.

The scores on PANSS are shown in **Table 4**. The ANOVA results revealed a significant difference in scores across the three social support subgroups at positive, negative, and comm symptoms domains, with lower scores being observed in higher social support groups.

**Table 4:** Scores on PANSS in Schizophrenia Patients, by Social Support Level (N=727).

PANSS	Overall Mean Score	Social Support			*f*	*P*
		Low (n=141)	Medium (n=336)	High (n=250)		
Positive	9.78 ± 3.81	10.53 ± 4.21	10.41 ± 3.97	8.52 ± 2.94	22.37	<0.01
Negative	9.68 ± 4.29	10.81 ± 5.19	10.04 ± 4.42	8.56 ± 3.15	15.24	<0.01
General psychopathology	19.12 ± 4.65	19.57 ± 4.52	19.91 ± 5.09	17.80 ± 3.73	16.16	<0.01
Average	3.44 ± 1.34	3.48 ± 1.85	3.54 ± 1.34	3.28 ± 0.91	2.77	0.06

Bold P-value represents statistics with statistical significance.

**Table 5** presented the associations of social support with symptom remission using several regression models. In the unadjusted model, social support was significantly associated with symptomatic remission [Odd’s Ratio (OR) = 3.20, 95% confidence interval: 2.45–4.18]. The association remained significant after being adjusted for patients’ characteristics (OR = 3.02, 95% CI: 2.30–3.97). The association remained statistically significant when family-related variables of patients were additionally added to the regression model (OR = 3.34, 95% CI: 2.53–4.43).

**Table 5:** The Association of Social Support with Symptom Remission in Schizophrenia Patient.

Model	*β*	OR	95%CI	*P* Value
Unadjusted model				
Social support	1.16	3.20	2.45–4.18	<0.01
Adjusted for patients’ characteristics				
Social support	1.11	3.02	2.30–3.97	<0.01
Age	0.08	1.08	0.80–1.47	0.61
Gender	0.68	1.97	1.24–3.15	<0.01
Residential area	-0.01	0.99	0.79–1.26	0.96
Education level	-0.25	0.78	0.61–1.01	0.06
Non married	0.01	1.43	0.71–2.90	0.05
Married	-0.38	0.69	0.37–1.25	0.18
Duration since diagnosis	-0.19	0.83	0.63–1.09	0.18
Adjusted for family-related variables				
Social support	1.21	3.34	2.53–4.43	<0.01
Family economic status	-0.10	0.91	0.66–1.26	0.56
Number of family members	0.06	1.06	0.84–1.35	0.61
Primary caregiver				
Parents	-0.07	1.24	0.54–2.82	0.67
Spouse	-0.01	1.32	0.59–2.98	0.97
Son/daughter	0.36	1.91	0.74–4.98	0.15
Caregiver’s education level	0.84	0.84	0.65–1.09	0.18

Bold P-value represents statistics with statistical significance.

## 4 Discussion

The current study investigated the symptomatic remission status quo among community-dwelling schizophrenia patients in Yingshan County during COVID-19. We found that a total of 727 patients enrolled in our study, 522 (71.8%) achieved symptomatic remission.

Previous studies found that the symptomatic remission rates in patients with schizophrenia ranged from 60%-91.5% before the pandemic (22, 23). In Bradford and Gaebel et al.’s study, the remission rate in schizophrenia patients varied from 87% to 89% in high-income countries and the remission rate showed an increasing trend (24, 25). In this study, we found that the symptomatic remission rate among community-dwelling schizophrenia patients during COVID-19 (71.8%) is relatively lower, compared to the rates reported by prior studies (22, 23).

The consensus that social support facilitates symptomatic remission of schizophrenia patients has been widely accepted. Therefore, we employed a face-to-face SSRS questionnaire to measure the status quo of social support among community-dwelling schizophrenia patients and further revealed its role in the decreased symptomatic remission rates during COVID-19. We further compared social support between remission and non-remission groups, in terms of overall scores, subjective support scores, and objective support scores. The results consistently showed that social support among the remission group was better than that of the non-remission group (all *P*<0.01, refer to **Table 2** for more details). Interestingly, we found that the overall social support scores (27.01 ± 6.67) in the study were lower relative to those (38.78 ± 7.60) reported by studies before the pandemic (19). This indicated that the pandemic had led to a decrease in social support, which may have contributed to a reduction in symptomatic remission rates among individuals with schizophrenia. Additionally, in this study, females accounted for 61% of the sample, which is higher than in previous research (26), which indicated that compared to males, females often receive lower levels of social support. This may also explain the relatively low level of social support observed in this study.

Social support was crucial for community-dwelling individuals with schizophrenia as it could significantly impact their well-being, quality of life, and ability to achieve symptomatic remission (27). Patients with schizophrenia often face social isolation, stigma, and discrimination, which could lead to feelings of loneliness, hopelessness, and despair. These negative experiences may further exacerbate their symptoms and decrease their motivation to engage in social activities. To investigate the relationship between social support and symptomatic remission in schizophrenia patients during the pandemic, we measured symptomatic remission using the PANSS scale, which was an internationally recognized standard endorsed by the RSWG. Our results were consistent with prior research, such as the study by Tominaga et al. (28), where lower PANSS scores (8.52 ± 2.94, 8.56 ± 3.15, and 17.80 ± 3.73, respectively) were associated with higher levels of social support and negatively associated with symptomatic remission.

However, the pandemic may have reduced social support for individuals with schizophrenia due to lockdowns, social distancing measures, and disruptions in health services, exacerbating the negative effects of social isolation and stigma on their well-being and recovery. The relatively lower social support during COVID-19, which ultimately leads to lower symptomatic remission rates among community-dwelling schizophrenia patients could be induced by multiple reasons. Firstly, the lockdown policy also reduced or completely halted interpersonal communication and community health services, which made it difficult for patients to maintain the balance between their positive and negative symptoms necessary for achieving symptomatic remission through higher social support levels and community health services during COVID-19. Secondly, disruptions in health services, such as the closure or reduction of primary mental health services, may have also contributed to a decrease in social support among individuals with schizophrenia during the COVID-19 pandemic, as they may have had reduced access to critical mental health care and medication management. Our study conducted a systematic and comprehensive assessment of social support during a crisis, which distinguishes it from previous studies.

Logistic regression was further employed to reveal the relationship between social support and symptomatic remission, controlling for patients’ characteristics and family-related variables. Consistent with previous studies (4, 14), we found that higher levels of social support were significantly associated with increased odds of achieving symptomatic remission (OR=3.02, 95% CI: 2.30–3.97, *P*<0.01), even after adjusting for potential confounding variables. The results reinforced our conclusions that the decreased remission rates could be partly attributed to worse social support during COVID-19. Providing extra care and social support during the pandemic was crucial for the symptomatic remission of individuals with schizophrenia. However, studies (29, 30) have shown that COVID-19 created various challenges for patients with schizophrenia, such as difficulty in medication delivery, low rates of regular medication adherence, and the closure of rehabilitation programs for several months. As a result, patients were subject to home quarantines or restrictions on rehabilitation programs, leading to lower levels of social support and a higher relapse rate among community-dwelling patients with schizophrenia.

The potential reason for the positive relationship between social support and symptomatic remission in individuals with schizophrenia was emotional support. However, during the COVID-19 pandemic, individuals with schizophrenia often lacked emotional support and were more likely to experience social stigma, as noted by Zhang et al. (31). Furthermore, they had limited support from society and their social circles, exacerbating these factors and resulting in a further decline in available social support for individuals with schizophrenia living in the community. Addressing these factors and enhancing social support networks for individuals with schizophrenia during the COVID-19 pandemic was crucial. Inadequate social support could increase relapse rates and perpetuate stigma and prejudice toward individuals with schizophrenia. Therefore, efforts to promote social support and reduce stigma during the pandemic were critical for the well-being and recovery of individuals with schizophrenia. By improving social support networks and reducing stigma, we could create a more supportive environment for individuals with schizophrenia, leading to better symptomatic remission and improved quality of life for schizophrenia patients the COVID-19.

Several limitations should be noted in this study. The first pertains to generalizability. Despite the cluster random sampling method employed in the study, our study area was limited to a certain county in Sichuan Province, China, which may weaken the generalizability of our findings. Second, as our data are self-reported, our results may be vulnerable to bias raised by measure errors. Thirdly, due to the cross-sectional design, it’s hard to a causal inference. Moreover, since we did not obtain data on the participants’ social support levels prior to the pandemic, longitudinal comparisons cannot be made. Despite these drawbacks, this research contributes to the sparse body of evidence on symptomatic remission among community-dwelling schizophrenia patients in China and the significance of social support during the COVID-19 pandemic.

## 5 Conclusions

The results reinforced our conclusions that the decreased remission rates could be partly attributed to worse social support during COVID-19. Thus, social support was recommended as an effective means of promoting symptomatic remission among community-dwelling individuals with schizophrenia during the pandemic. By strengthening social support networks and providing effective mental health services, we could enhance symptomatic remission and recovery of community-dwelling schizophrenia patients, ultimately improving their quality of life.

## Data availability statement

The original contributions presented in the study are included in the article/supplementary material, further inquiries can be directed to the corresponding author.

## Ethics statement

The studies involving humans were approved by the Institutional Ethics Review Board (IERB) of the Psychosomatic Hospital Affiliated to North Sichuan Medical College in June 2021. Written informed consent for participation in this study was provided by the participants/participants' legal guardians/next of kin before they began the questionnaire. The studies were conducted in accordance with the local legislation and institutional requirements.

## Author contributions

LY: Formal analysis, Writing – review & editing, Writing – original draft. HL: Writing – original draft, Resources. QP: Writing – review & editing, Data curation, Methodology. XT: Writing – original draft, Writing – review & editing, Funding acquisition, Project administration.
